# The impact of tinnitus distress on cognition

**DOI:** 10.1038/s41598-021-81728-0

**Published:** 2021-01-26

**Authors:** P. Neff, J. Simões, S. Psatha, A. Nyamaa, B. Boecking, L. Rausch, J. Dettling-Papargyris, C. Funk, P. Brueggemann, B. Mazurek

**Affiliations:** 1 Department of Psychiatry and Psychotherapy, University of Regensburg, Regensburg, Germany; 2 University Research Priority Program ‘Dynamics of Healthy Aging’, University of Zürich, Zürich, Switzerland; 3 Tinnitus-Zentrum, Charité - Universitätsmedizin, Berlin, Germany; 4 Terzo Institute, ISMA AG, Sonneberg, Germany

**Keywords:** Human behaviour, Neuroscience, Neurology, Signs and symptoms, Epidemiology, Translational research

## Abstract

Tinnitus is the chronic perception of a phantom sound with different levels of related distress. Past research has elucidated interactions of tinnitus distress with audiological, affective and further clinical variables. The influence of tinnitus distress on cognition is underinvestigated. Our study aims at investigating specific influences of tinnitus distress and further associated predictors on cognition in a cohort of n = 146 out-ward clinical tinnitus patients. Age, educational level, hearing loss, Tinnitus Questionnaire (TQ) score, tinnitus duration, speech in noise (SIN), stress, anxiety and depression, and psychological well-being were included as predictors of a machine learning regression approach (elastic net) in three models with scores of a multiple choice vocabulary test (MWT-B), or two trail-making tests (TMT-A and TMT-B), as dependent variables. TQ scores predicted lower MWT-B scores and higher TMT-B test completion time. Stress, emotional, and psychological variables were not found to be relevant predictors in all models with the exception of small positive influences of SIN and depression on TMT-B. Effect sizes were small to medium for all models and predictors. Results are indicative of specific influence of tinnitus distress on cognitive performance, especially on general or crystallized intelligence and executive functions. More research is needed at the delicate intersection of tinnitus distress and cognitive skills needed in daily functioning.

## Introduction

Tinnitus is the perception of a chronic auditory phantom sound, usually in the form of a high-pitched tone, ringing or noise, in the absence of any objective external sound source^1,2^. It is defined as chronic after a continuous presence of one year^3^. Prevalence rates of individuals experiencing tinnitus are roughly between 12 and 30%^4,5^ with 1% to 3% suffering considerably from the condition^6^. In most cases, tinnitus is caused by hearing loss or damage to the peripheral auditory system which in turn leads to maladaptive plasticity of the auditory pathway and the brain^7^. Several partly conflicting models about tinnitus generation and chronification exist while the ‘hard problem’ of pinpointing the persistent phantom sound perception to neural markers remains a conundrum^8^.

In order to better understand emotional and behavioral consequences of tinnitus, a plethora of descriptive and experimental research has been performed in the last 20 years. The interactions of tinnitus with other clinical measures, especially affective dimensions like depression^9^, anxiety^10^, and stress^11^ have been thoroughly studied. Established knowledge points at considerable comorbidity rates and shared neural resources with tinnitus^12,13^. Further research avenues aimed at investigating tinnitus consequences with regards to auditory perceptive functions like directional hearing^14–16^ or speech perception^17–19^. Results of these studies are indicative of tinnitus-specific reductions in auditory performance which in consequence may have a limiting influence in daily life functioning of tinnitus patients. Yet, a recent study questions these observations and theories by demonstrating no differences between tinnitus patients and healthy controls in 32 out of 36 audiometric tests^20^ Hearing loss and auditory functioning are especially relevant in the context of aging where evidence points to related cognitive decline and related central neuroplastic changes^21,22^.

Former studies investigated the relationship between tinnitus and cognition. Two recent reviews identified up to 18 experimental studies studying various cognition aspects like attention, working memory, executive functions, processing speed, verbal abilities and intelligence^23,24^. Most studies applied between-subjects designs examining differences in cognitive performance between individuals with and without tinnitus whereas just a few studies reported exploratory correlations with tinnitus measures. Given our study design investigating differential influences on cognition within the tinnitus population, we here focus on correlational analyses and not group contrasts with healthy or clinical controls. Results of reviewed correlational studies are generally indicative of reduced cognitive performance in tinnitus, especially with respect to attention, processing speed and executive functions (e.g.,^25,26^) while there are also reports of null or conflicting findings^25,27–29^. Moreover, it has been discussed if high-frequency hearing loss should be rather considered as the cause of observed effects of lower cognitive performance, especially working memory^29,30^.

The study at hand aims at extending previous research by testing a clinical sample of tinnitus patients with respect to their cognitive skills in a set of standardized tests, namely a intelligence test (‘Mehrfachwahl-Wortschatz-Intelligenztest’, MWT-B^31^) and trail making tests (TMT, -A and -B^32^). In a next step, the predictive effects of tinnitus-related, demographic and clinical variables on the measured test performance are modeled by means of a machine learning regression approach (elastic net). In particular, we are interested in the putative amount of negative influence of tinnitus distress as measured by the Tinnitus Questionnaire (TQ^33^) on performance in the 3 cognitive tests. A few former studies have included TMTs as part of neuropsychological batteries in the context of tinnitus^27,34,35^. Gabr et al.^34^ found reduced tests performances for both TMT-A and TMT-B in tinnitus patients compared to healthy controls whereas^27,36^ reported worse TMT performance of tinnitus patients compared to norm values. Vanneste et al.^35^ reported positive correlations (indicative of a negative influence on cognitive performance) of tinnitus distress, loudness, duration, but not hearing loss with TMT performance. Looking at general, crystallized or verbal intelligence we identified several studies most of which were testing these concepts by means of the Mini-Mental State Examination (MMSE^27,35,37^). Generally, lower MMSE scores were observed in all studies comparing tinnitus groups to controls or norms. In addition, effects were less pronounced in TMT assessments. Andersson et al. ^38^ applied a subtest from the Wechsler Adult Intelligence Scale measuring verbal ability in their study which identifies as the single study relevant to our study design deploying the MWT-B. With regards to (verbal) intelligence, this former study reported a trend difference between groups with a lower mean score in the tinnitus group. Authors noted that the intelligence scale was assessed to control for possible differences between groups and thus not part of the main analysis pipeline.

Given the aim and scope the study, the applied methods, the study sample, and the results of previous studies, we assume that tinnitus distress negatively affects the performance in standardized cognitive tests. While some previous research identified first evidence for these assumptions, our study surpasses previous studies with both a dedicated study design and the application of statistical methodology able to thoroughly disentangle predictors of cognitive performance. The latter is especially critical, as former study mostly performed group contrasts or exploratory correlational analyses. To the best of our knowledge, this is the first study which investigates the influence of tinnitus distress and other tinnitus-related variables on cognitive performance including a comprehensive set of control variables. Concretely, we hypothesize that tinnitus distress negatively predicts performance in all of the 3 cognitive tests. Besides that, we expect negative as well as positive influences of age and education on cognitive performance, respectively, which are well-established relations in the general population and therefore supposedly also present in our tinnitus study sample. Furthermore, hearing parameters are included to differentiate tinnitus and general hearing-specific influences on cognition. Finally, the inclusion of psychological and emotional variables will allow for further differential assessment of cognitive performance within the tinnitus population.

## Methods

### Participants

146 out-ward tinnitus patients (74 female) were recruited for participation in this study which was performed in two consecutive sessions between 2017 and 2019. Inclusion criteria were chronic subjective tinnitus with a minimum duration of 3 months (Tinnitus aurium ICD10:H93.1), a biological age between 18 and 75 years, and written informed consent. Individuals with severe physical or psychological conditions, tumors in the head region, epilepsy, Morbus Meniére, ototoxic medication, substance abuse, chemotherapy, severe hearing loss ($\ge$ 85 dB averaged over frequencies between 0.5 and 4 kHz in the better hearing ear according to WHO 4 guidelines), or participation in a different study were excluded from this study.

Participants were informed about the aim, scope and proceedings of the study, were given the opportunity to ask questions, and finally signed the informed consent agreement after reading it. The study data at hand comprises baseline measurements of a longitudinal intervention study. In the first session, the clinical assessment including audiometry was performed. Behavioral and psychometric measurements were assessed by paper and pencil in the second session. Detailed participants characteristics are listed in Table 1. This study was approved by the local ethic commission of the Charite - Universitätsmedizin Berlin (No. EA1/114/17) and informed written consent was received from all participants. All experiments and procedures were performed in accordance with relevant guidelines and regulations.

### Audiometry

Audiometry was performed in the facilities of the audiological department of the clinic. All measurements were performed in a sound-proof booth with dedicated and calibrated devices.

#### Pure tone audiometry

Pure tone audiometry (PTA) was measured using a clinical audiometer (AT900, Auritec, Germany) and air-conduction headphones (DT 48, Beyerdynamic, Germany). Measurements were performed for the two ears and the frequency range between 0.25 and 10 kHz in octave or semi-octave steps. PTA scores were averaged over both ears and all frequencies for statistical analyses.

#### Speech-in-noise

In order to test participants’ ability to detect and correctly identify words of a speech signal in background noise, a German speech-in-noise (SIN) test was performed (‘Freiburger Wörtertest’^39^). A monophonic speaker was used as the audio source which was centrally positioned 1 meter away in front of the listener. Three group of words with identical recognition difficulty were selected and presented at 65 dB SPL with speech babble as the noise background at either 0, 55, or 65 dB, respectively. The application of speech babble as background noise is the single modification of the original SIN protocol^39^. Correct hits in percent (i.e., unambiguous identification of the word), averaged for the two ears from the most difficult condition (65 dB speech babble), were included in the statistical models.

### Psychometry

We assessed various tinnitus-related and general tinnitus-unspecific psychometric measures including tinnitus distress, anxiety and depression, stress levels, and general psychological well-being. While tinnitus distress or affective disorders are commonly checked in tinnitus research and clinical practice^40^, stress levels and more general psychological well-being have been reported less frequently^11,41,42^.

#### Tinnitus questionnaire

A German version of the Tinnitus Questionnaire (TQ^33,43^ was used to assess the amount of subjective tinnitus distress. TQ covers tinnitus distress facets like emotional distress, cognitive distress, intrusiveness, sleep disorders, somatic complaints, and auditory perceptual difficulties on a three-point Likert scale. It comprises 52 items and the maximal total score is 84 points. For clinical classification TQ scores can be graded in 4 levels from mild to very severe. TQ can be considered as the established extensive questionnaire on tinnitus distress with high validity, reliability, and many international adaptations^44^. The total score was used for statistical analyses of this study.

#### Hospital anxiety and depression scale

Hospital Anxiety and Depression Scale (HADS^45,46^) was deployed to inquire anxiety and depressive symptoms of the study sample in a German adaptation of the questionnaire^47^. HADS assesses 14 items on a four-point Likert scale from 0 to 3 resulting in a total score of 42, or 21 for the two subscales anxiety and depression. For the statistical analyses of this study, we used the total scores of the two subscales.

#### Perceived stress questionnaire

The perceived stress questionnaire (PSQ) assesses momentary subjective stress perception^48^. It comprises 20 items in the form of short statements about current stress-related emotions and thoughts. PSQ is further subdivided into 4 subscales covering worries, tension, demands, and joy separately. The total score ranges from 0 to 100 with 50 being the cutoff for high level perceived stress. For the current study, a short-form German adaptation of PSQ was used^49^ and its total score subjected to statistical analyses.

#### ICD-10 symptom rating questionnaire

The ICD-10 Symptom Rating (ISR) questionnaire is established on the basis of symptom lists of the well-known ICD-10 clinical categorization system and measures various psychological aspects like depression, anxiety, obsessive and compulsive disorders, somatoform and eating disorders, and a supplementary scale covering a variety of syndromes^50,51^. ISR consists of 29 items scored between 0 and 4 (0 = not true, 4 = very true) and covers 6 syndrome scales. For the study at hand, we used a German adaptation of the ISR 2.0^50^ and its total score for statistical analyses.

### Cognitive tests

In order to test for general (crystallized) intelligence and specific cognitive performance in processing speed, (visual) attention, and executive functions, two standardized test batteries were deployed as described in the following.

#### Multiple choice vocabulary test

We used the German Multiple choice vocabulary test (MWT-B^31^) to assess general and crystallized intelligence. The participant is instructed to strike through a known word from a row containing multiple words (1 known colloquial or scientific word and 4 similar fictional words). The test comprises 37 items (i.e., rows) with increasing difficulty. Correct items are then summed up to a total score of 37 and standardized to IQ scores^31^. Number of correct items was the measure used for our statistical analyses.

#### Trail making test

A German version of the Trail making test (TMT) was used (TMT-A and -B^32^). The first task (TMT-A) requires a participant to connect a sequence of 25 consecutive numbers as fast as possible. The second task (TMT-B) complements TMT-A with consecutive letters of the alphabet which have to be connected in addition to the numbers (e.g., 1, A, 2, B etc.). The outcome variable is the time needed to complete the task (seconds) including potential errors, which was chosen as the dependent variables for our modeling purposes. TMT-A is designed to test cognitive processing speed whereas TMT-B examines executive functioning^32^. In clinical or general assessment practice, both tests are usually conducted and a ratio can be calculated to be used as a composite score. Given our interest in differential aspects of cognition, statistical models were build for TMT-A and TMT-B independently.

### Statistical analyses

R software was used to analyze the behavioral data and to produce the figures (R version 3.4.2; R Foundation for Statistical Computing, Austria^52^) and the packages “psych”, “tidyverse”, “glmnet” and “caret”. For descriptives we report absolute numbers (counts), mean and median values, standard deviations (SD), and extreme values (i.e., minima, maxima). Spearman correlations were performed on continuous variables included an reported in the supplemental materials for exploratory reference (Fig. S1). The level of statistical significance was set to $\hbox {p}< 0.05$ for all analyses.

#### Elastic net regression

In order to test our hypotheses we performed analyses calculating 3 models with either MWT-B, TMT-A, or TMT-B as dependent variables. Elastic net, a generalization of Ridge and Lasso regression, accounts for collinearity by penalizing the coefficients in the model either by shrinking their values or by setting them to zero, thus providing a suitable tool for settings with many predictors, and for performing automatic predictors selection^53^. A n-fold cross validated elastic net was used to estimate the lambda (i.e., a tuning parameter used to minimize the sum of squared error) over 11 different alpha values ranging from 0 (i.e., RIDGE regression) to 1 (i.e., Lasso regression). As a requirement of the glmnet package, predictors were scaled and centered. This procedure renders the coefficients interpretable and enables to rank coefficient importance by the relative magnitude of post-shrinkage coefficient estimates. The model with minimized mean squared error was selected for each of the dependent variables and reported in this analysis. Performance metrics of the models (i.e., root-mean-square error (RMSE) and standardized coefficients (R2)) are reported in the results section alongside predictor coefficients.

## Results

Descriptive statistics are listed in Table 1.

**Table 1:** Participant characteristics.

Variable	Mean	SD	Median	Min	Max	Range
Age (years)	59.4	7.41	60	29	74	45
Education (level)	2.55	1.1	2	0	4	4
Hearing loss (both ears, dB)	29	7.08	28.69	11	48.8	37.8
TQ (total score)	33.1	16.2	30.5	8	80	72
Tinnitus duration (months)	180	128	152.5	16	728	712
PSQ (total score)	30.4	19.6	25.83	1.7	100	98.3
ISR (total score)	0.64	0.56	0.48	0	3.08	3.08
HADS anxiety (total score)	7.84	3.1	7	3	16	13
HADS depression (total score)	5.34	4.94	4	0	21	21
SIN (both ears, hit rate %)	15.52	8.07	14	4	41	37
MWT-B (total score)	29.16	3.82	30	19	37	18
TMT-A (total score)	35.19	11.34	32.74	16.94	83.06	66.12
TMT-B (total score)	81.42	32.21	74.06	31.84	218.34	186.5

*SD* standard deviation.

**Figure 1:**
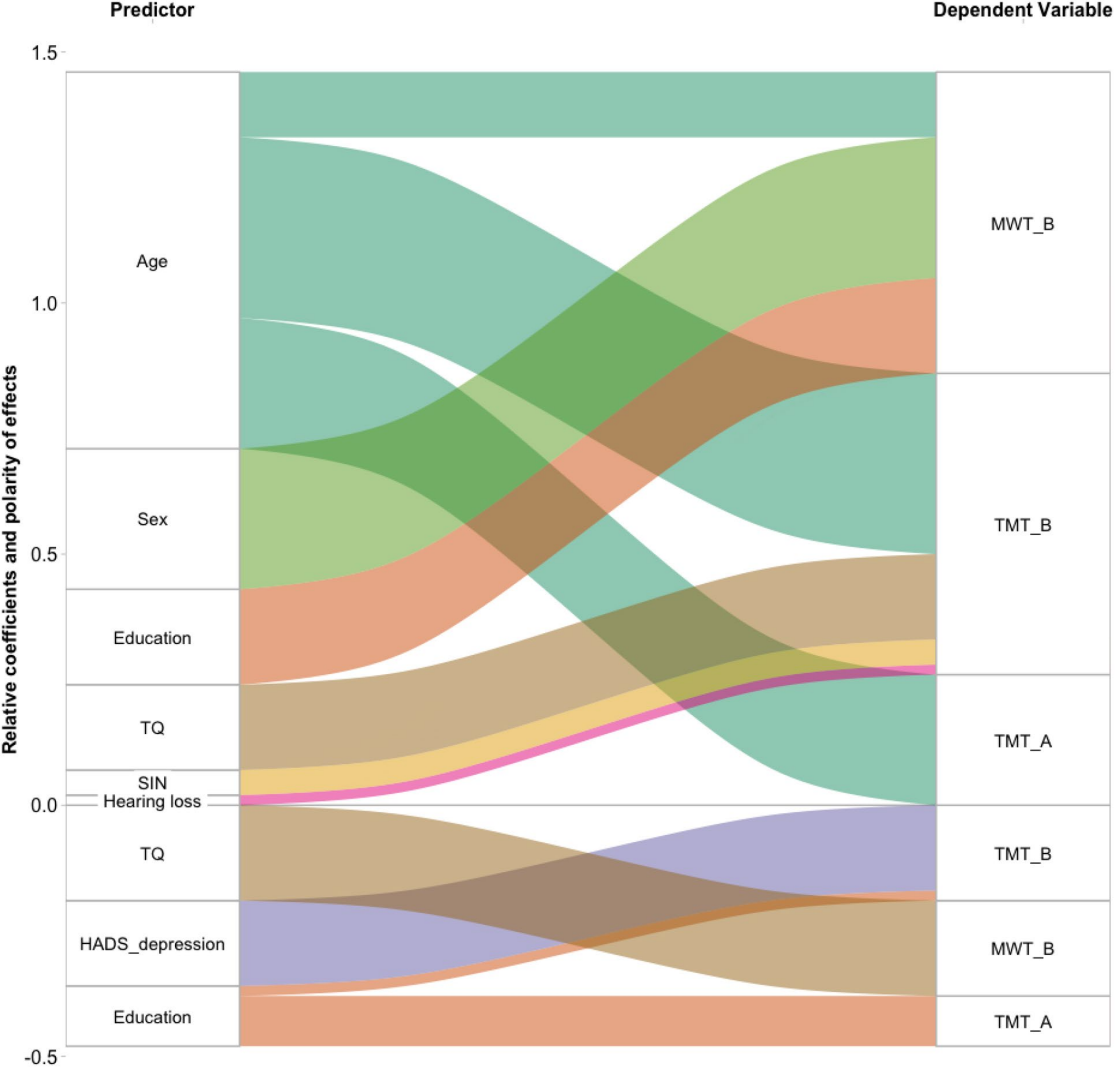
Sankey plot of main results of the 3 regression models. Predictors are plotted on the left and represent the source of the ribbons. Dependent variables of the 3 models are plotted on the right and serve as the targets of the ribbons. Relative influence of standardized predictors is coded by the width of the ribbon and the source as well as target boxes on the y axis. Polarity of coefficients is visualized by their position above or below the x axis at 0. Coefficient values can be retrieved from Table 2.

We applied 3 elastic net cross-validated regression models with the various predictors as independent variables and the 3 cognitive measures MWT-B, TMT-A, and TMT-B as dependent variables. The cross-validation process including convergence on the final model is plotted in Fig. S2 of the Supplemental Materials. Detailed results for the 3 models including standardized coefficients are reported in Table 2 and Fig. 1.

**Table 2:** Model parameters and coefficients of the 3 models.

Model	MWT-B	TMT-A	TMT-B
R2	0.22	0.19	0.04
RMSE	0.91	0.87	0.75
(Intercept)	$-$ 0.45	$-$ 0.01	0.02
Age	0.13	0.26	0.36
Sex	0.28	0	0
Education	0.19	$-$ 0.1	$-$0.02
Hearing loss	0	0	0.02
SIN	0	0	0.05
TQ	$-$ 0.19	0	0.17
HADS depression	0	0	$-$ 0.17
Tinnitus duration	0	0	0
PSQ	0	0	0
HADS anxiety	0	0	0
ISR	0	0	0

Coefficients are visualized in Fig. 1. RMSE = root-mean-square error.

For the model probing influences on MWT-B, we can report an R2 of 0.22 denoting that 22% of the variance in MWT-B can be explained by the included predictors. Most notably, age, sex, and education had a small positive influence on MWT-B scores while TQ exerted a small negative influence.

The second model elucidating influences on TMT-A performance (i.e., time to complete the test; R2 = 0.19) resulted in a small positive effect for age, which comes as no surprise given the generally declined processing speed related to normal aging. In addition education was found to exert a small negative influence on test completion duration. TQ was not found to be a valid predictor for TMT-A test performance.

In model 3 (R2 = 0.03) again age was the most prominent and pronounced predictor of TMT-B scores (medium effect size). Notably, the R2 is relatively small compared to the other models, theory and former findings, which might be explainable by the large variability of the TMT-B scores. In line with the findings of model 1 with respect to a negative influence of tinnitus distress on cognitive performance, TQ exerted a small positive influence on test completion duration. Furthermore, we found a small positive influence of education on TMT-B performance, which complements the expected effects of education on cognitive performance. Finally, SIN and hearing loss showed a small positive influence whereas depression scores were found to be negative predictors of test completion duration.

## Discussion

This study aimed at investigating the predictive role of tinnitus distress and other tinnitus-related variables on cognitive performance assessed with tests on general (or crystallized) intelligence, processing speed, visual attention, and executive functions.

Tinnitus distress seems to negatively affect general or crystallized intelligence and executive functions while not influencing processing speed. This finding is novel given the nature of applied methodologies and the within-group analysis in contrast to between-group findings of lower intelligence^27,35,37^, various impaired cognitive abilities except long-term memory^54^, increased risk of Alzheimer’s and Parkinson’s disease^55^, and lower TMT performance^27,34–36^ in tinnitus patients. In some studies these behavioral findings were further corroborated by neurophysiology, mostly in the form of longer latencies of standard event-related EEG components in auditory oddball tasks^54,56^. Yet, while not directly applicable, results from theses studies fit our hypothesis with respect to intelligence as measured by the MWT-B while we could not find any effect for the TMT-A. Notably, the observed effects are not driven by age or any other of the included variables given the rigid methodology of the elastic net regression approach accounting for collinearity by penalizing predictors. The applied novel methodology thus can be considered a strength of our study compared to previous research on tinnitus and cognition. Furthermore, an expected pattern of results emerges with age predicting cognitive test scores in all analyses in the theorized direction, namely increasing recognized word count (crystallized intelligence) and time to complete the TMTs (reduced processing speed, visual attention and executive functions). This observation is in line with established knowledge about the role of aging in developing higher levels of crystallized intelligence and reducing fluid aspects of cognition like processing speed, working memory and attention^57^. Besides these hypothesized outcomes, the absent relation between tinnitus distress and performance in one of the TMT tests in our study is noteworthy as it contradicts findings of previous studies. It has to be considered that these studies mostly used between-subjects designs comparing tinnitus patients with healthy controls^23,24,58^ and thus are more sensitive to such an effect. Currently, we can only speculate about our null-findings with respect to TMT-A scores which were commonly more pronounced in group differences in the former studies than differences in intelligence. Mean scores are in range of population norms^32^ and data distribution looks normal. In general, we assume that negative influences of tinnitus distress on cognition can be explained by shared and partly conflicting (neural) resources of tinnitus and cognition systems. In the case of the MWT-B scores, this resource conflict might also cover working memory. The positive influence of depression (HADS) on TMT-B performance is an unexpected result of our study, which we cannot explain in any specific manner at this point. The same is true for higher MWT-B scores in women, which might stem from a respective bias in the study population. Notably, this effect of biological sex can only reported for the MWT-B model. To the best of our knowledge, no former study studied this specific interaction of SIN, tinnitus distress, and cognition. Therefore, our finding of a weak influence of SIN on cognitive performance (here: TMT-B task completion time) should be tested against healthy controls with respect to reported influences of SIN on executive functions, especially working memory and inhibition^59^. In any case, observed effects of better SIN performance in relation to longer TMT-B test completion time in this study are counterintuitive and not compatible with current models or data on resource conflicts between the two domains. As with the effects of depression scores and to a lesser extent sex, we surmise that this result is most likely an artifact of the statistical analyses, possibly related to inherent properties of the specific study sample (selection bias) and should thus not be overinterpreted. Neither HADS anxiety nor the ISR or PSQ scores were relevant predictors for cognitive performance in the 3 tests. In combination, these findings point at the absence of any influence of affective, psychological or stress disorders on cognitive performance in tinnitus patients. Furthermore, it allows to conclude that decreased cognitive performance is more likely to be driven by tinnitus distress and (related) hearing loss, and the expected differential influences of age and education level.

Our questionnaire and test battery comprised a wide variety of methods to test aspects of tinnitus, psychological health and cognition. It is certainly advisable to extend and refine applied methodology for future studies to be able to draw more solid and valid conclusions from resulting data. In addition to the current measures, we propose the inclusion of an additional layers of psychological phenotypization putatively influencing pathological dynamics^60^. This is especially true for personality^61–63^, which was never specifically studied in the context of tinnitus and cognition. Cognitive tests are furthermore naturally linked to neuropsychology, which implies the use of respective neuroscientific methods. Some previous studies combined behavioral and neurophysiological methods for the study of cognition, mostly attention, in tinnitus (e.g.,^35,54,56,64–66^). Various task-related or group-specific maladaptive alterations of neurophysiology were reported for tinnitus patients in these studies. None of these studies investigated neural correlates of the tests which we found to be influenced by tinnitus distress in our study.

Taken together, results of this study are novel in that 1) a solid negative influence of tinnitus distress on cognitive performance can be reported and 2) the decreased TMT performance in tinnitus patients observed in former case-control studies might be linked to tinnitus distress in our data. Moreover, the results from this study are clearly in favor of tinnitus-specific effects on cognition while affective, stress, or psychological aspects are unrelated to cognitive performance in our study population.

### Limitations

We also report some limitations for our study which may influence current conclusions and inform follow-up research. First, a greater number of methods and more differentiating measures of cognitive performance should be considered for future studies. In our and other former studies, short-form and general cognitive tests were mostly used, which is especially true for intelligence. Extensive tests on differential aspects of intelligence and other cognitive abilities would allow to better study causalities and mechanisms in tinnitus and cognition. Related to that, better control for psychosocial variables (e.g., socioeconomic status, social embeddedness, quality of life, and personality) would increase the validity of found effects and mechanisms. The absence of any influences of predictors other than age and education on TMT-A scores may reflect that the test was too easy and thus not sensitive enough for the differential predictors in the study population at hand. Future studies should therefore consider more feasible instruments to probe processing speed. A major limitation of our study is the absence of high-frequency hearing loss as well as analyses thereof to rule out putative influences on cognition^29,30^. Yet, looking at our results with an averaged hearing loss as predictor, hearing loss seems to exert a small negative influence on TMT-B scores related to working memory as theorized in the former studies while the relation with high-frequency hearing loss can not be calculated with our data. Follow-up research should therefore strive for the inclusion of high-frequency hearing loss data in such that well-balanced analyses of different hearing profiles become possible. Furthermore, hearing data could be complemented with (additional) supra-threshold tests and measures of otoacoustic emmissions. Direction of effects assumed have to withstand scientific scrutiny in a series of studies,as always advisable in predictive regression analyses. Given our hypotheses, data and results, we assume that the directionality of our effects is valid while we currently can not completely rule out the opposite. Some studies and theories specifically focused on cognitive influences on (the generation of) tinnitus distress or annoyance and thus did assume an opposite directionality of effects^26,67–69^. We tested a rather larger sample of n=146 clinical tinnitus patients, yet, a selection bias with variable distributions atypical in the general population might distort results. Found effects should be replicated across centers and tinnitus subpopulations in large numbers^70^. Finally, longitudinal studies would be needed to test observed effects with regards to their stability over time and possible susceptibility to changes in related variables.

## Conclusion

In a clinical population of tinnitus patients, tinnitus distress seems to negatively influence cognitive performance. This is especially true for general or crystallized intelligence as elicited by MWT-B and for executive functions as measured by TMT-B. To a lesser extent, hearing loss seem to exert a similar effect on executive functions while age and education showed the expected, well-established effects on all 3 cognitive measures. The novel results of this study underline the importance of tinnitus-related factors in cognition. More research is necessary for both a better basic understanding of how tinnitus affects cognition but also of related implications on real life functioning. Future studies could furthermore consider an extended set of behavioral, audiological, and neuroscientific measures to be able to resolve the delicate interactions between tinnitus and cognition.

## Supplementary Information

Supplementary Figures.
