# Supplementary material for: The impact of tinnitus distress on cognition
Source: Sci Rep. 2021 Jan 26;11:2243. doi: 10.1038/s41598-021-81728-0 (PMC7838303; doi:10.1038/s41598-021-81728-0)
Supplement: Supplementary file 1 — Supplementary Figures. [file 41598_2021_81728_MOESM1_ESM.pdf]

Supplemental materials

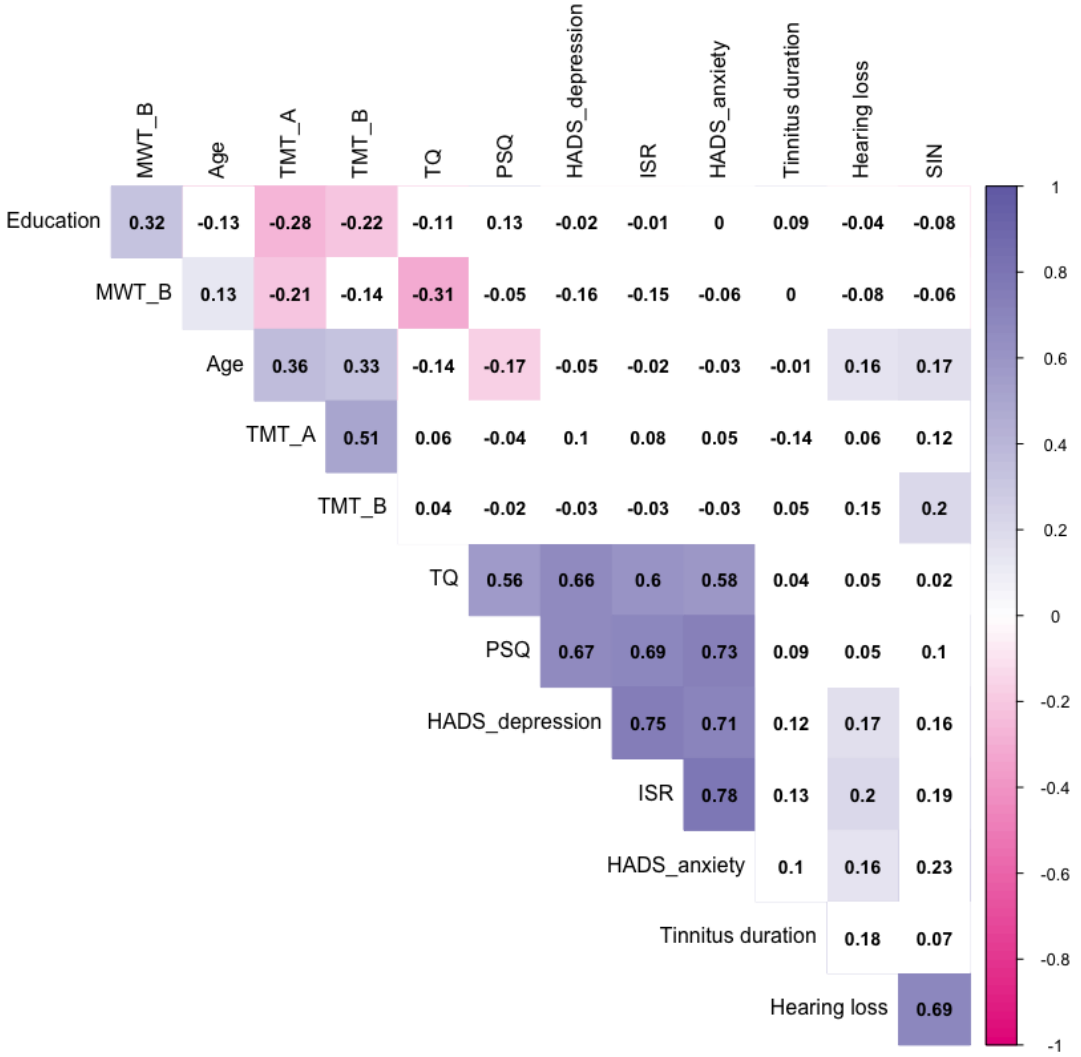

Figure S1. Correlation matrix of variables of interest for exploratory purposes. Spearman correlations were used. Colored tiles are indicative of significant correlations (p<0.05) and correlation strength coded in color saturation (positive = blue, negative = red). Correlations are sorted with hierachical clustering.

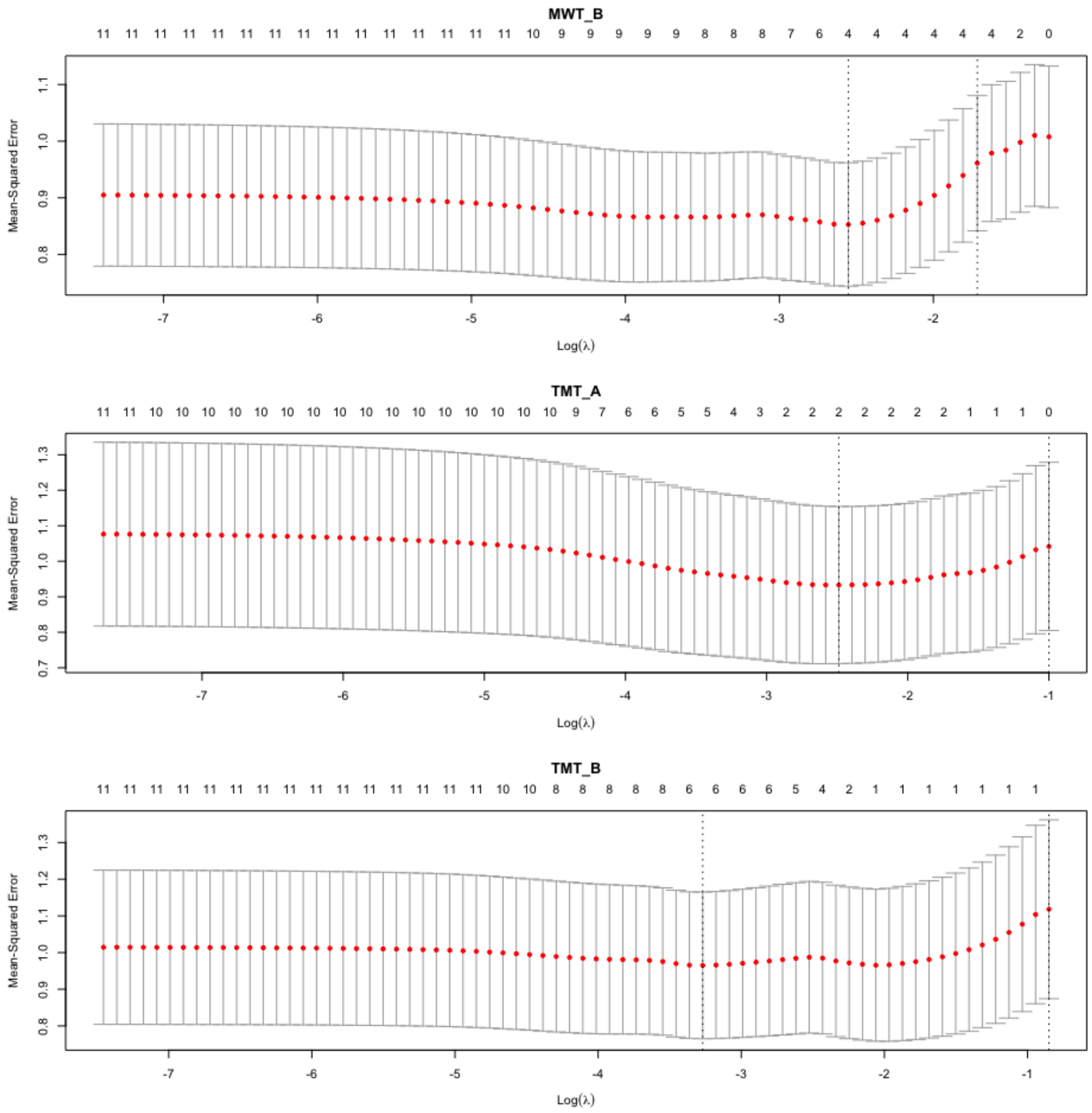

Figure S2. Cross-validation of all models.. Leave-one-out cross-validation for n observations was performed where MSE was minimized for model selection (left dotted vertical line). Numbers on top of the plot indicate number of predictors.
